# The 2022 and 2023 Emergency Medicine Residency Match: A Cautionary Tale

**DOI:** 10.7759/cureus.38601

**Published:** 2023-05-05

**Authors:** Mary Lewis, Kathleen Williams, Joshua Timpe, Samuel Corbo, Morgan Wilbanks, Alisa K Hayes

**Affiliations:** 1 Emergency Medicine, Medical College of Wisconsin, Milwaukee, USA

**Keywords:** soap, emergency medicine residency, residency, the match, emergency medicine

## Abstract

Introduction

The emergency medicine (EM) match has undergone significant shifts in 2022 and 2023. While variation in specialty fill rates is expected over time, EM programs noted a significant increase in open positions starting in 2022. Utilizing National Resident Matching Program (NRMP) data over a 10-year period, we identified significant deviations in the emergency medicine match.

Methods

Shewhart control charts were used to plot the match results over time. A 10-year sample was used to establish the baseline value. From this value, the upper and lower control limits were established. Residency program expansion, decreasing applicant numbers, and changing applicant types were evaluated to detect any non-random changes to the process.

Results

While the number of EM PGY-1 positions added over time was within the expected range, both the number of unmatched positions and the change in the number of total US MD applicants were outside of this range and are considered to be "out of control."

Conclusion

It is not yet clear which contributing causes may underlie this sudden change. Several potential etiologies exist, including mismatches in supply and demand for positions, changes in perceptions of the specialty, the effects of COVID-19, and changing workforce needs. Historically similar experiences affecting other specialties, including anesthesia and radiation oncology, are analyzed. Potential solutions for returning to the necessary and usual success of the emergency medicine specialty match are explored.

## Introduction

Prior work has demonstrated the ebb and flow of competitiveness in certain medical specialties over time [1-3]. The emergency medicine (EM) match, while previously stable, saw a significant alteration in activity in the 2021-2022 cycle, which continued into the 2022-2023 match cycle. Despite warnings of this potential [4], EM saw a significant increase in unfilled match positions. In each of the previous ten years, EM had a match fill rate greater than 99% [5]. Prior to the additional match offerings through the Supplemental Offer and Acceptance Program (SOAP), the 2022 match cycle left 219 postgraduate year one (PGY-1) EM positions unfilled, with a match fill rate of 93%. This trend continued to worsen in the 2023 match cycle, leaving 555 unfilled PGY-1 EM positions open and a match-fill rate of 81.6%.

We analyzed the 2022 and 2023 EM match data to determine if the variation in match outcomes had deviated significantly from expected outcomes. We analyzed the total number of positions, the total number of unfilled positions, the type of applicants (MD, DO), and the location of the medical school (United States vs. international). While it may be challenging to identify the root cause of this dramatic change in the match-fill rate, prior similar experiences in other specialties have identified contributing factors such as the increase in the number of residency positions despite stagnant application numbers, disruption of specialty perception, and changes in workforce needs. We additionally review each of these factors with the goal of adding objective data to the discussion on how the specialty can mitigate further increases in unfilled spots and a decline in competitiveness. We hope the experience in EM can be utilized as a cautionary example and help other specialties avoid similar situations.

## Materials and methods

Utilizing data from the National Resident Matching Program (NRMP) and the Electronic Residency Application Service (ERAS), we analyzed trends over a 10-year period. We assessed the number of available PGY-1 EM spots offered in each match cycle, as well as the number of applicants and the number of unfilled PGY-1 EM positions.

Shewhart control charts were used to plot the match results over time. Control charts are used to assess if a process is under control over time with repeated sampling. A process that is under control is within the limits that can be followed over time. If outputs occur that are outside of the expected range and are statistically unlikely, this can provide an alert to assess the causes of the deviation. This graphical representation has been used to illustrate significant deviations in the residency match process in other specialties in a previous study [3].

The baseline accepted value is established by an average of historical values. A 10-year sample was used to establish the baseline value. From this value, the upper and lower control limits are established. The control limits are calculated by taking the accepted baseline value and adding and subtracting the process standard deviation to find the upper and lower control limits, respectively. A process is considered to be under tight control when the deviation over time falls within 2⋅𝜎 (process standard deviation). A measurement in the process is considered egregiously out of control when the deviation is above 3⋅𝜎 (process standard deviation).

This type of analysis has been used in engineering, business, and healthcare processes to evaluate quality [6]. Analyzing process variation can assist in differentiating common cause variation versus special cause variation. The Nelson rules are a method to determine if a measured variable is "out-of-control" or a non-random condition [7]. These eight rules can be applied, and if met, they can indicate when a potential "out-of-control" situation has arisen.

## Results

**Figure 1:**
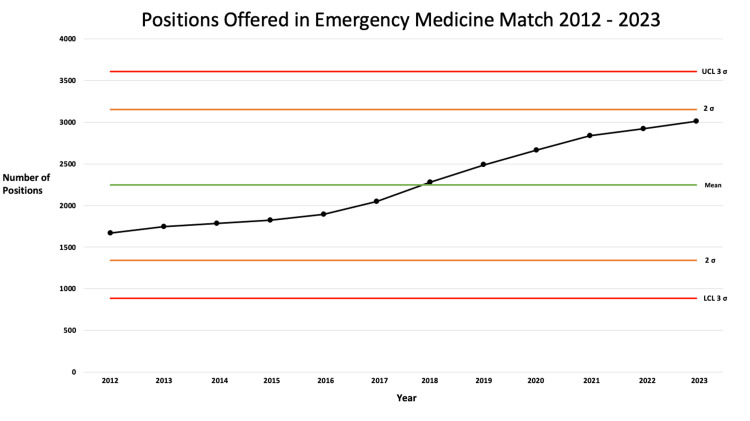
Control chart: positions offered in the emergency medicine match 2012–2023 UCL: upper control limit, LCL: lower control limit, σ: standard deviation

While the total number of spots increased significantly over a 10-year period, the range stayed within the expected control range based on the mean (Figure 1). By Nelson's rule 3, the continuously increasing values verify that an upward trend in PGY-1 EM positions is occurring.

**Figure 2:**
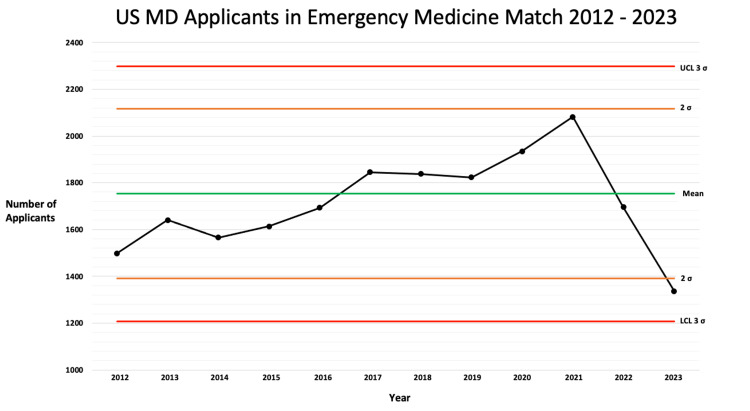
Control chart: US MD senior applicants to the emergency medicine match 2012–2023 UCL: upper control limit, LCL: lower control limit, σ: standard deviation

The number of US MD applicants to emergency medicine has been variable in recent years, with the most recent match in 2023 indicating the decline, particularly of US MD applicants, is no longer under usual control and on the brink of being "out of control" (Figure 2). Rule five states that if two points in a row are more than two standard deviations from the mean, then the process is out of control. This same finding is not present in osteopathic and international graduate application numbers, where rates remain under control and show an increasing trend in recent years for international graduates.

**Figure 3:**
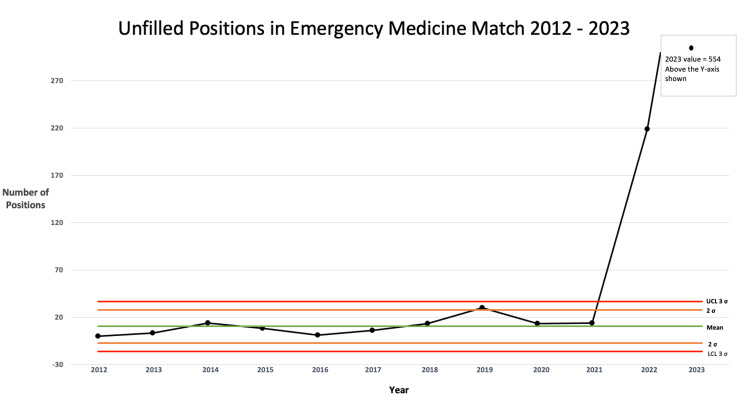
Control chart: unfilled positions prior to SOAP in the emergency medicine match 2012-2023 UCL: upper control limit, LCL: lower control Limit, σ: standard deviation, SOAP: Supplemental Offer and Acceptance Program

Lastly, the number of unfilled positions in emergency medicine, both in 2022 and 2023, is egregiously out of control (Figure 3). Applying Nelson rule 1 indicates that there is a significant deviation. Rule 1 states that if one point is more than three standard deviations from the mean, the process is grossly out of control.

## Discussion

Mismatch in applicants and training positions

Emergency medicine is a relatively young field that developed as a response to a need for emergency care first identified during the World War II era. Despite decades of development and the establishment of EM as a specialty in 1979, there was a perceived shortage of EM physicians over the first several decades of its existence. In 1995, there was a call for new EM programs to open in response to this need [8]. Positions and the subsequent supply of emergency medicine residents then increased rapidly with the creation of new programs, resulting in a 74% increase in training positions between 2008 and 2019 [9,10]. More than 40 previously American Osteopathic Association (AOA)-accredited programs have transitioned to Accreditation Council of Graduate Medical Education (ACGME) accreditation by 2020 [11].

Similar to EM, the specialty of anesthesia had a call for more physicians to enter the field in the 1980s, and as a result, GME graduates quadrupled in the time period between 1984 and 1993 [12]. Job market availability did not keep up; thus, several years later, the field saw a downtrend in the match. This was believed to be due to concerns about a lack of security in the job market noted by medical students and highlighted in the lay media [13]. This resulted in a match as few as 44% of positions being filled in 1995 [14].

Radiation oncology was more recently affected by a surplus in residency positions compared to the number of trainees. Despite identifying a mismatch between expanding programs and a downtrend in applicants in 2015, the number of PGY-1 positions continued to expand, with a solid surplus identified in the 2019-2020 cycle. Fifteen percent of the radiation oncology spots were filled via SOAP in that cycle. There continued to be a surplus of residency positions in the 2021 cycle, with only 12 of the total 15 PGY-1 residency positions and only 138 out of 173 PGY-2 positions being filled in the main match cycle [2].

Regardless of previous specialty examples and workforce projection needs, emergency medicine programs have continued to expand in both size and number. The volume of expansion did not mirror changes in applicant numbers. There was a mismatch between the number of training positions, which continued to grow, and the number of applicants. The increase in training programs included new EM programs established and sponsored by national contract groups and national hospital networks, as well as previous osteopathic programs that joined the ACGME and many existing programs that increased in size. This change was not driven by identified workforce needs. Some geographic areas were more impacted by the osteopathic program transitions and the opening of new programs. National groups began to recommend a more cautionary approach to this expansion, specifically highlighting the market forces motivating the creation of new residency programs [15].

The mismatch in positions between applicants alone does not fully account for the events of the match cycle these past two years. Additional considerations would include factors affecting the interest in the specialty among students, including changes in the applicant's view of the specialty and concerns about future job satisfaction and security.

Perception of specialty

The perception of emergency medicine by potential applicants may also be evolving. Emergency medicine has long been considered a specialty offering a controlled lifestyle, allowing "personal time free of practice requirements for leisure, family, and avocational pursuits and control of total weekly hours spent on professional responsibilities" [16]. Work-life balance has shown increasing importance among both Millennials and Generation Z. A 2022 survey by Deloitte et al. found work-life balance to be the top priority when making a job or career choice [17]. These cultural shifts may be contributing to previously down-trending fill rates in specialties such as surgery, where lifestyle was one of the most frequently cited factors for students not selecting general surgery as a career [18]. Conversely, controllable lifestyle specialties, at least as a group, continue to be highly sought after by US medical school graduates.

While emergency medicine may offer more lifestyle control than other specialties, it also has among the highest rates of burnout when compared to other specialties. A recent systematic review found the prevalence of burnout among EM physicians to be as high as 71.4% [19]. This has the potential to disincentivize students seeking a controlled lifestyle career away from EM and towards other specialties. High rates of burnout may also separate emergency medicine from other identified controlled lifestyle specialties. In surveys, younger Millennials as well as Generation Z, who will soon be taking over as the majority of EM trainees, have shown an increasing value for job stability and certainty [20]. While we were unable to find literature specifically addressing student perceptions of burnout and its impact on specialty choice, this well-known trend in emergency medicine, along with recent concerns about the EM job market, is likely contributing to decreased interest among US medical students.

The lay media also plays a role in public perception and likely influences potential applicants. Very recent media articles highlight the bad news and do not tell the whole story of the specialty, including the numerous positives [21]. These attention-grabbing negative headlines are likely contributing to poor perceptions of the specialty without presenting the whole story [22].

COVID-19 pandemic

Further complicating the future of emergency medicine, the COVID-19 pandemic has certainly changed the perception of the specialty. However, it is not clear whether this will be a positive or negative shift. Early in the pandemic, front-line healthcare workers, including EM, were painted as "healthcare heroes," especially in the media. This status elevation may have contributed to an increase in applications in the cycle immediately following the start of the pandemic. Conversely, as the pandemic has evolved, it may be significantly impacting the specialty's perception in a negative way. Higher risks associated with front-line work, along with the increasing strain on emergency department resources and a further increase in the already high burnout rates since the start of the pandemic, may be further painting emergency medicine in an unfavorable light. Specifically, a survey of New York City (NYC) medical students found that COVID-19 may have discouraged students from considering emergency medicine [23]. While this may not be generalizable, especially given the course of COVID-19 through NYC early in the pandemic, it certainly warrants monitoring. Likely, it will take several years before the lasting impact of COVID-19 on the perception of emergency medicine is completely understood. This, combined with recent concerns raised about the EM job market, may compound hesitancy among EM applicants.

Workforce needs

A highly publicized emergency medicine workforce analysis, published in 2021, highlighted these pulls in supply and demand as well as projected attrition and predicted that there would be a surplus of 9,314 physicians by 2030 if the field continues to grow at the same rate [24]. In addition, there is an increasing concern that a surplus of emergency medicine physicians will affect physician salaries and competitiveness in the market [4,9,10]. Although a subsequent analysis suggests the surplus may be overstated, there remains significant uncertainty about the future workforce [25]. This may further drive down the interest of prospective medical students, many of whom have significant outstanding loan repayments.

Negative articles about the specialty, either in the lay media or within publications of EM, could drive qualified applicants away from training in the specialty. The field of anesthesia has seen this happen. Anesthesia experienced a dramatic increase in residents applying for attending positions after the number of graduates quadrupled over a 10-year period. Subsequently, they experienced a significant decline in applicants to the field. There was a 56% decline between 1995 and 2000 [5]. This was believed to be in part due to warnings heeded by popular media regarding the competitiveness of the specialty and the difficulty in finding jobs [14]. It is important to consider the message emergency medicine is providing regarding the future of the specialty.

An additional factor at play is the use of advanced practice providers (APPs) in numerous emergent settings, including emergency departments and urgent care facilities. APPs have been utilized in the emergency department increasingly since the mid-2010s. There are concerns regarding the expanding role of APPs within EM, as APPs can obtain similar positions in the workforce at a lower cost than physicians [26]. As a comparison, the number of Certified Nurse Anesthetist (CRNA) trainees increased during the downtrend in physician trainees. More recently, CRNAs have been increasingly utilized in the hospital setting, with some hospitals even replacing their anesthesiologists with CRNAs [27]. In 2021, the American Association of Nurse Anesthetists offered members the option to call themselves nurse anesthesiologists, which resulted in condemnation by the American Society of Anesthesiologists due to concerns pertaining to patients being misinformed about the training and skill set of those providing care [28]. Advanced practice providers can be valuable members of a healthcare team and may be willing to fill in the gaps by providing care in settings where minimal care is available; however, without appropriate boundaries, market forces could encourage the overutilization of APPs to the detriment of our physician trainees.

The EM workforce is not homogenous, with a geographic mismatch in the available physician workforce most notable between urban and rural settings. Numerous rural EM facilities are staffed by physicians and advanced practice providers who are not board certified in EM. Despite the growing workforce, there is a continued need for EM residency-trained physicians in these communities. EM physicians comprise 63.9% of the workforce in urban populations and only 44.8% of the workforce in rural communities, with this discrepancy only widening in recent years [29]. Furthermore, there is an increase in the number of ED visits in rural communities. This highlights an area of unmet demand in the EM workforce.

Current state and future directions

We saw a further downtrend in applications to EM in 2023, resulting in a further increase in open positions, more than doubling in size. It will be important to continue monitoring these trends in emergency medicine and to find ways to match quality medical students into the field of emergency medicine, ensure that residency programs are meeting important standards to train residents, and ultimately match those applicants that will have successful careers in emergency medicine.

Preference signaling was introduced into the EM match for the 2023 cycle. This supplement to the ERAS application has been shown to potentially improve the ease of match for programs and their interest in applicants. In 2017, otolaryngology saw a significant decrease in the number of applicants [5]. This was thought to be related to increasing barriers to applying and changes in application requirements, including a talent assessment [3,30]. Otolaryngology now uses preference signaling, which is one factor that could have contributed to the stabilizing number of applicants over the past several years [5]. As the specialty of EM grapples with the possibility of large numbers of unfilled programs, preference signaling may become a powerful tool for programs to select applicants more likely to rank them highly.

Given the concern for potential future career options, as the number of residency spots continues to expand, it will be important that we continue to provide opportunities for EM physicians to fill other needs within the healthcare system. One such avenue would be to have a more deliberate approach to training our graduates to practice in settings where there exists a significant shortage of emergency medicine physicians, such as rural communities [29]. The rise of some subspecialties like wilderness medicine, which teaches skills for working in remote and austere environments, as well as the increase in residency programs providing rural exposure and rotations, may help fill this gap.

Additionally, there could be consideration of branching into other fields. This has been done by specialties such as trauma surgery. In the early 2000s, trauma surgery was thought to be going by the wayside. Trauma surgeons redefined themselves as specialists in all emergency surgeries and surgical critical care [31]. Different avenues for emergency physicians include prehospital care, home health, telemedicine, and further specialization in other niches within medicine through fellowship training in ultrasound, critical care, sports medicine, wilderness medicine, toxicology, and global health. Fellowships within these areas are becoming more widespread and available to residency graduates.

## Conclusions

The emergency medicine residency match is "out of control." The most obvious cause of the unprecedented number of unfilled programs and open residency positions is a mismatch between supply and demand. An increasing trend of additional residency positions paired with a decline in applicants, particularly among US MD senior medical students, has resulted in the current match outcomes. Certain states and regions, especially those with higher numbers of previous osteopathic residencies and those with larger numbers of new programs, have been more significantly affected. Applicant perceptions of the future of the EM specialty continue to evolve as a new generation of physicians enters the workforce. US medical student perceptions are likely to have been influenced by the lay media and scientific publications. Factors presented include the COVID-19 pandemic, pre-pandemic forecasts of a surplus of EM physicians, and the increasing role of advanced practice providers in the specialty. The current change in applicant numbers will have a profound impact on the EM specialty and the patients we serve. In highlighting these recent changes, we hope to inform the discussion on how best to move forward. Ongoing attention and analysis are required to ensure that the safety net of our healthcare system remains intact. Medical students must be provided with accurate information regarding workforce needs as well as the benefits and challenges of any specialty they may be considering. Despite this latest challenge, EM physicians will adjust, rally, recruit, and continue to show up for our patients. EM remains an integral part of care for all types of patients and offers a flexible and long career for a diverse trainee population.
